# Comparative study on alkaloids and their anti-proliferative activities from three *Zanthoxylum* species

**DOI:** 10.1186/s12906-017-1966-y

**Published:** 2017-09-12

**Authors:** Yongqiang Tian, Chunyun Zhang, Mingquan Guo

**Affiliations:** 1 Key Laboratory of Plant Germplasm Enhancement and Specialty Agriculture, Wuhan Botanical Garden, Chinese Academy of Sciences, Moshan, Wuchang, Wuhan, 430074 China; 2 Graduate University of Chinese Academy of Sciences, Beijing, 100049 China; 3 The Sino-Africa Joint Research Center, Chinese Academy of Sciences, Wuhan, 430074 China

**Keywords:** *Zanthoxylum* alkaloids, LC-UV-ESI-MS/MS, Anti-proliferative activity, Fingerprinting analysis

## Abstract

**Background:**

Alkaloids have been considered as the most promising bioactive ingredients in plant species from the genus *Zanthoxylum*. This study reports on the compositions and contents of the *Zanthoxylum* alkaloids (ZAs) from three *Zanthoxylum* species, and their potential anti-proliferation activities.

**Methods:**

An HPLC-UV/ESI-MS/MS method was established and employed to analyze the alkaloids in different *Zanthoxylum* extracts. The common and unique peaks and their relative contents were summarized and compared to evaluate the similarity and dissimilarity of the three *Zanthoxylum* species. Meanwhile, inhibitory activity tests to four carcinoma cell lines, i.e., stomach tumor cells (SGC-7901), cervical tumor cells (Hela), colon tumor cells (HT-29) and Hepatic tumor cells (Hep G2), were carried out in vitro to evaluate the bioactivities of the ZAs.

**Results:**

Seventy peaks were detected in the crude total alkaloid samples, and 58 of them were identified. As a result, 13 common peaks were found in the extracts of all the three *Zanthoxylum* species, while some unique peaks were also observed in specific species, with 17 peaks in *Z. simulans*, 15 peaks in *Z. ailanthoides* and 11 peaks in *Z. chalybeum*, respectively. The comparison of the composition and relative contents indicated that alkaloids of benzophenanthridine type commonly present in all the three *Zanthoxylum* species with high relative contents among the others, which are 60.52% in *Z. ailanthoides*, 30.52% in *Z. simulans* and 13.84% in *Z. chalybeum*, respectively. In terms of activity test, Most of the crude alkaloids extracts showed remarkable inhibitory activities against various tumor cells, and the inhibitory rates ranged from 60.71 to 93.63% at a concentration of 200 μg/mL. However, SGC-7901 cells seemed to be more sensitive to the ZAs than the other three cancer cells.

**Conclusion:**

The alkaloid profiles detected in this work revealed significant differences in both structures and contents among *Zanthoxylum* species. The inhibitory rates for different cancer cells in this study indicated that the potential anti-cancer activity should be attributed to quaternary alkaloids in these three species, which will provide great guidance for further exploring this traditional medicinal resource as new healthcare products.

## Background

Various plants of the genus *Zanthoxylum* have been used not only as anti-flooding roundworm and tooth pain in ancient China, but also as flavoring and antiseptic additives. The main chemical components of these plants from this genus, such as *Z. nitidine*, *Z. bungeanum*, *Z. simulans* and *Z. armatum*, could be classified as volatile oils, lignins, flavonoids and alkaloids [1–4], in which alkaloids from the genus *Zanthoxylum* (ZAs) were considered to be the most potent anti-tumor ingredients. For some known ZAs, the existing studies showed several mechanisms to further explain the anti-tumor activities, e.g., decarine, 6-acetonyldihydrochelerythrine and zanthocapensine induced colon cancer cells (HCT116) apoptosis by activating casepase-3 pathways [5]; Sanguinarine, Chelerythrine and Chelidonine exhibited anti-proliferative activity against mouse lymphocytic leukemia cells (L1210) by damaging leukemia DNA [6]; nitidine and angoline showed inhibitory activity against carcinoma cells by suppress c-Src/FAK [7] and IL-6/STAT3 [8] pathways. Although the pharmacological studies have boosted the progress in exploring this medicinal resource, the investigation of the chemical basis of different plants of the genera *Zanthoxylum*, and the screening of new potential bioactive compounds from this genus are still hotspots for further exploring new anti-tumor drugs or healthcare products from the medicinal plants of this genus.

The conventional way to characterize ZAs usually involves the isolation of pure compounds and subsequent structures determination based on their NMR spectra [9], which is not only time and labor consuming, but also inefficient and insensitive to some ZAs in the characterization process, especially to those in very minute amount. To circumvent these limitations, a series of instrumental methods have been applied for the quantitative and qualitative analysis of ZAs, such as HSCCC (high-speed Countercurrent Chromatography), CE (Capillary electrophoresis), GC-MS, LC-MS, and LC-MS-NMR [10–18]. Due to the high performance in separation and sensitivity of alkaloids in the positive mode during the electro-spray ionization, LC-ESI-MS has become one of the most powerful methods for the analysis of alkaloids from plants of these species [19, 20].

In this work, an HPLC-UV-ESI-MS/MS method was developed to analyze the crude alkaloids from *Z. ailanthoides*, *Z. simulans* and *Z. Chalybeum*. Combining with chemical fingerprinting analysis of the crude extracts, the corresponding anti-proliferative activity tests were also conducted for the same samples against hepatic cancer (Hep G2), gastric cancer (SGC-7901), colon cancer (HT-29) and cervical cancer (Hela) cells in order to correlate the contribution of different types of alkaloids to their diverse anti-proliferative activities. This work may lay the groundwork for the further understanding of anti-cancer compositions from these three *Zanthoxylum* species.

## Methods

### Chemicals and reagents

Formic acid, ammonium, methanol, triethylamine, chloroform, acetic acid, trichloroacetic acid and dimethyl sulfoxide (DMSO) were of analytical grade and purchased from Sino-pharm Chemical Reagent CO. (Beijing, China). Water for HPLC and LC-MS was prepared with EPED (Nanjing Yeap Esselte Technology Development Co., Nanjing, China). The medium, DMEM, Fetal calf Serum and Pancreatic enzymes, for Carcinoma cell culture were obtained from Wuhan Feiyi Science and technology Co. (Wuhan, China).

### Plant materials

Fresh plant materials of two *Zanthoxylum* species (*Z. simulans,* and *Z. ailanthoides*) were collected from Wuhan Botanical Garden in April, 2015. Fresh stem bark of *Z. chalybeum* was collected from Kenya. The authentication and identification of the specimens was kindly assisted by the taxonomist (Prof. Guangwan Hu) of Key Laboratory of Plant Germplasm Enhancement and Specialty Agriculture (Wuhan Botanical Garden), Chinese Academy of Sciences. All of voucher specimens (No. 001–003) were deposited in the herbarium of the Key Laboratory. After dried at 30 °C, the plant materials were crushed and stored at room temperature until use.

### Plant extraction

After being immersed in 70% ethanol (with 0.5% HCl) for 12 h, plant materials of these three species were ultrasonically extracted for 30 min in thrice. The extracts were combined and condensed to form dark residues. Then, the resulted residues were dispersed in water, and extracted with chloroform to remove chlorophyll after the pH of water phase was adjusted to 9–11. Finally, chloroform phase was combined and concentrated to afford crude alkaloids.

### HPLC and LC-MS conditions

A Thermo Accela 1250 HPLC equipped with an auto-sampler and a UV-visible detector (Thermo Fisher Scientific, San Jose, CA, USA) was employed for the analysis of crude alkaloids. A 10 μL sample was analyzed on a Phenomenex Kinetex column (2.6 μm, C18, 100 × 2.1 mm) at 25 °C. The flow rate was 0.2 mL/min and the chromatograms were recorded at the wavelength of 280 nm. 0.5% formic acid solution (adjusted to pH = 4.5 by ammonium, A) and acetonitrile (B) were selected as mobile phase, and the gradient was set as follows: 0–25 min, 5–20% (B); 25–40 min, 20% (B); 40–55 min, 20–35% (B); 55–65 min, 35–80% (B).

For ESI-MS/MS analysis, a Thermo Accela 600 HPLC system with both UV detector and TSQ Quantum Access MAX mass spectrometer (Thermo Fisher Scientific, San Jose, CA, USA) was used for the LC-MS analysis in the positive mode. The mass condition was set as follows: mass range from 200 to 800 Da; Spray Voltage, 3.0 kV; Capillary temperature, 350 °C; Sheath gas pressure, 40 psi; Aux gar pressure, 10 psi.

### Qualitative and quantitative analysis

The alkaloids were identified based on the comparison of detected MS/MS spectra with reported literatures, MS/MS spectra of reference standards and their fragment pathways. Finally, they were semi-quantified by the relative peak areas in the HPLC chromatograms.

### In vitro anti-proliferative activity assay

Four Human cancer cell lines i.e., stomach tumor cells (SGC-7901), cervical tumor cells (Hela), colon tumor cells (HT-29) and hepatic tumor cells (Hep G2), obtained from the China Center for Type Culture Collection (CCTCC), were used for anti-proliferative activity tests with the crude extracts using Sulforhodamine B (SRB). These cancer cells were incubated into 96-well plate under 5% CO_2_ at 37 °C for 24 h and treated with crude ZAs at a concentration of 200 μg/mL in three duplicates, and DMSO was used as negative control. To make sure the security of the cells, the final concentration of DMSO was less than 0.1%. After incubation for 72 h, the supernatant was discarded, and 100 μL 10% trichloroacetic acid (TCA) was added into the wells and washed after the cells were fixed for 1 h. SRB was used to dye cells, and 1% acetic acid was used to remove the excess amount of SRB. The optical density (OD) values were determined at 565 nm [21, 22]. The inhibitory rate (%) equals to (ODC-ODT)/ODC × 100%, where ODT and ODC are the OD values of the blank and the alkaloids extracts, respectively.

## Results and discussion

### Optimization of HPLC conditions

The previous phytochemical investigation of ZAs indicated that the structure diversity and complexity may cause much difficulty in HPLC separation. To get better chromatograms of the crude alkaloids, the HPLC conditions were optimized for the analysis of the extract of *Z. ailanthoides.* Using one-variable method, the chromatographic column was firstly optimized by successively separating the crude extract on phenomenex kinetex C18 (100 × 2.1 mm, 2.6 μm) and Phenomenex ODS column (150 × 2.00 mm, 5 μm). As a result, phenomenex kinetex C18 (100 × 2.1 mm, 2.6 μm) was found to have higher column efficiency, and thus selected for the further sample analysis. Considering the significant effect of mobile phase and additives on alkaloids separation and peak shape, the mobile phase conditions based on the reported systems, such as 0.1% formic acid - 0.1% formic acid acetonitrile, 50 mM ammonium acetate-methanol, 0.5% acetic acid and 0.1% triethylamine-acetonitrile, 0.1% formic acid-acetonitrile and 0.5% formic acid (pH adjusted to 4.5 by ammonia)-methanol-acetontrile [13, 15, 23–25], were also investigated. As preferred, 0.5% formic acid (pH adjusted to 4.5 by ammonia)-acetonitrile was found to be the best mobile phase system for the analysis of the crude alkaloids extracted from *Z. ailanthoides.* At last, the final optimized conditions were achieved for the fingerprinting analysis of ZAs from these three species.

**Fig. 1:**
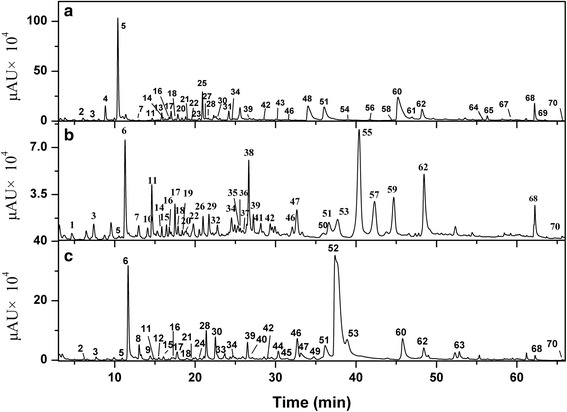
Fingerprinting analysis of *Z. ailanthoides* (**a**), *Z. simulans* (**b**) and *Z. chalybeum* (**c**)

Under the optimized HPLC conditions, the crude alkaloids of these three species (*Z. ailanthoides*, *Z. simulans* and *Z. chalybeum*) were analyzed, in which the chromatograms were shown in Fig. 1. It was observed that 70 peaks in the three extracts have been well separated and detected, indicating that the operation conditions adopted were suitable for the analysis of ZAs. Meanwhile, a preliminary study of the chromatograms revealed that the alkaloids compositions and contents exhibited significant differences among the three species.

### Identification of Zanthoxylum alkaloids

**Fig. 2:**
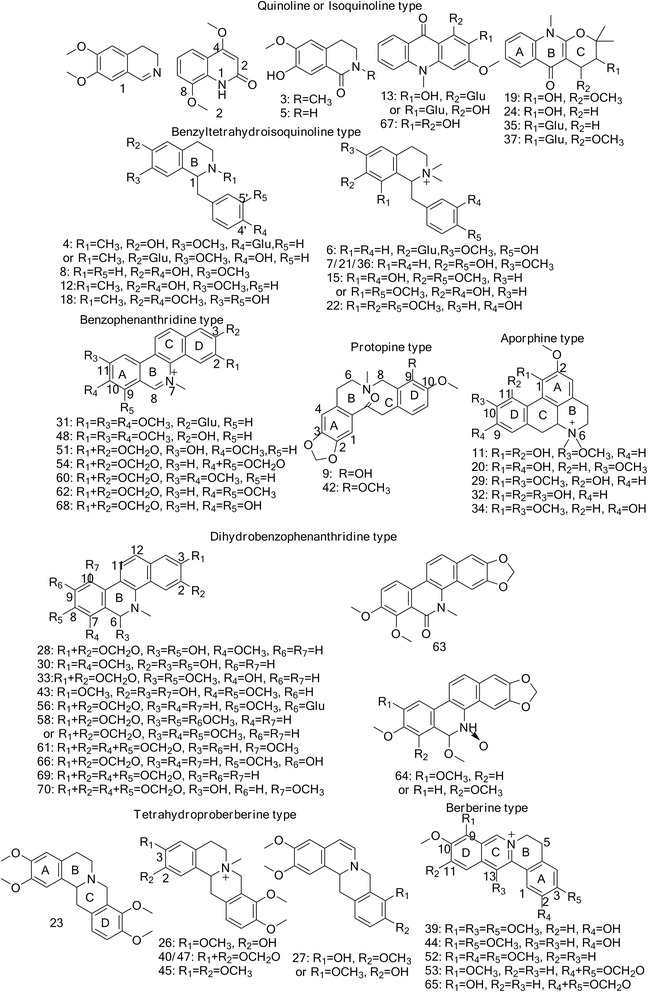
Chemical structures of ZAs identified from *Z. ailanthoides*, *Z. simulans* and *Z. chalybeum*

Among the detected peaks, 58 alkaloids were identified based on the reported literatures, standard spectra or fragment pathways, 31 of which (labeled as “b” in Table 1) were identified by comparing the MS/MS spectra with reported literatures. Based on the identification, the structures of the 58 ZAs were shown in Fig. 2. To the best of our knowledge, 15 of the ZAs (labeled as “a” in Table 1) were firstly reported in the genus *zanthoxylum*, and six (peaks **4**, **6**, **13**, **31**, **35** and **37**) were found to be alkaloids glycoside. Since different types of ZAs have distinguishing fragment pathways in ESI-MS/MS, we classified the alkaloids into eight types to simplify the structures interpretations, i.e., quinolone and iso-quinolone type, benzyltetrahydroisoquinoline type, benzophenanthridine type, dihydrobenzophenanthridine type, berberine type, tetrahydroproberberine type, protopine type and aporpine type [26–28]. The interpretations on their MS/MS data were discussed as below.

**Table 1:** MS/MS data and identification of detected ZAs

Peak NO.	Rt (min)	M^+^/ [M + H]^+^ (m/z)	MS/MS spectrum	Identification
1	4.67	192	192,177,162,148,133	3,4-dihydro-6,7-dimethoxy-isoquinoline^a^
2	6.11	206	206,190,173,162,145,132	Edulitine
3	7.39	208	208, 176,160,145,117,115	Thalifoline
4	8.83	462	300, 269,237,209,197,175,143, 115, 107	7-methoxy-2-methylhigenamine-4′-glucoside^a^or7-methyoxy-2-methylhigenamine-6-glucoside^a^
5	10.92	194	194,177,162,149,145,134,117, 89	Des-N-methyl-thalifoline^a^
6	11.67	476	476,314, 283,269,268,252,237, 209, 189,174,166,137, 107	Magnocurarine-6-glucoside^a^
7	12.99	314	314,269,237, 209, 166,137, 107	Magnocurarine^b^
8	13.01	286	286,269,254,237, 160,143,107	7-methoxylhigenamine^b^
9	14.09	356	356,338,323,206,188,179,151,149	Hunnemannine^b^
10	14.41	504	504,459,442,428,414,401,386,357,306,212,207	Unidentified
11	14.61	342	342,297,282,265, 237,222,219,207, 191	Magnoflorine^b^
12	12.28	300	300, 269,254,237, 160,143,107	7-methoxy-N-methylhigenamine^a^
13	15.85	434	434,272,255,237,161,143,123,107	2-dihydroxy-3-methoxy-10-methyl-9-acridone-1-glucoside^a^or1-dihydroxy-3-methoxy-10-methyl-9-acridone-2-glucoside^a^
14	16.26	328	328,312,234,205,190,178,163,148,135,119,91	Unidentified
15	16.43	344	344,301, 269,239,207, 143,137	(−)-isotembetarineor (−)-xylopinidine
16	17.31	390	390, 330, 285,269,254,241,237, 181	Unidentified
17	17.76	312	312,297,268,252,240,226,192,177,121	Unidentified
18	17.87	330	330,207,192,177, 175,143,137, 115	Reticuline^b^
19	18.16	290	290,272,242,218,201,188,175,160,132	4′-methoxy-ribalinine^a^
20	18.36	342	342,282,265,250,237,233,222,205,191	Laurifoline^b^
21	19.56	314	314,269,237, 209, 166,137, 107	Magnocurarine^b^
22	19.92	358	358,298,283,267,239,221,206,189,174,158,151,137,122,115,105	8-methoxy-isotembetatrine^a^
23	20.53	356	356,340,289,206,192, 177,163,149	Tetrahydropalmatine^b^
24	20.58	260	260,242,200,188,176,160,134	Ribalinine
25	20.92	540	540,313,287,272,268,253,249,201,175,163,143,131	Unidentified
26	20.98	356	356,192,177, 151,149	N-methyltetrahydrocolumbamine^b^
27	21.35	340	340,325,291, 190,161,149,119	2-methoxy-5-dehydro-scoulerine^a^
28	21.63	352	352,337, 322,319,308,291,280,266	Buegenine
29	21.71	356	356, 311,296,279,264, 248,236, 219,192,177	Menisperine^b^
30	25.59	354	354,339,322,311,308,293,280,262,252	2-dimethoxy-3-hydroxybeugenine^a^
31	22.77	512	512,350,335,307,292,264	Unidentified
32	23.07	328	328,283,251,238,208,191,159,143,121	10-demethyl-magnoflorine or avicine^b^
33	23.31	366	366,350,334, 322,318,294,276	6-methyl-5,6-dihydrofagaridine^b^
34	24.7	356	356,311,296,280,265,253,237,219, 192,177	Xanthoplanine^b^
35	25.33	422	422,260,242,224,210, 188	Ribalinine 3′-glucoside^b^
36	25.6	314	314,269,237, 209, 166,137, 107	Magnocurarine^b^
37	25.92	452	452,290,272,257,242,240,239,225,218	4′-methoxy-ribalinine-3′-glucoside^b^
38	26.66	680	680,662, 547,435, 322,227,209,114	Unidentified
39	27.24	368	368,352,338, 324,310,307,292,278	10-hydroxy-2,3,9,12-tetramethoxy-jatrorrhizine^a^
40	27.11	354	354,338,190,175,149	N-methylcanadine or its isomers^b^
41	28.18	342	342,282,266,251,235,206,189,174,158, 135,121	Unidentified
42	29.04	370	370,352,320,290,206,188,165,149	α-allocryprotopine^b^
43	30.05	384	384,368,352,337,335,320,306,292	6,10-dihydroxyl-3-demethyldihydrobenzophenanthridinium^b^
44	30.33	338	338,322,308,294,280,279,265	Jatrorrhizine^b^
45	31.44	370	370, 308,206, 190,173,162,145	N-methyltetrahydropalmatine
46	32.69	292	292,274,256,226,202,188,172,144, 117	Unidentified
47	33.13	354	354,338,190,175,149	N-methylcanadine or its isomers^b^
48	34.03	350	335,334,320,307,292,277,264,236	Fagaronine
49	34.75	354	354,338,322,310,294, 190	Unidentified
50	35.89	246	246,231,216,202,188,184,156	Haplopine^b^
51	36.63	334	334,319,304,291,276,262	8-O-demethylchelerythrine^b^
52	37.38	352	336,322,320,308, 292, 278,264	Palmatine^b^
53	38.88	336	336,320,306,304,292,278,262	Berberine^b^
54	38.96	332	332,317,304,287,274,261,246,215	Sanguinarine^b^
55	40.38	490	490,288,272,246,240	Unidentified
56	41.66	498	498,336,321,305,293,264,250	8-O-demethyl-dihydrochelerythrine −9-glucoside^a^
57	42.3	468	468,288,270,260,225,242,218,190, 164	Unidentified
58	44.69	380	380,364,349, 320,306,292,278,249	8-methoxy-dihydronitidine^a^ or 8-methoxy-dihydrochelerythrine^a^
59	44.71	322	322,304,286,271,256,232,217, 201,189,174,159,145	Unidentified
60	45.22	348	348,332,318,304,290	Nitidine^b^
61	45.22	364	364,348,334,332,320,304,290,246	Dihydrochelirubine^b^
62	48.2	348	348,332,318,304,290	Chelerythrine^b^
63	52.84	364	364,348,334, 316,306,289,276	Oxychelerythrine^b^
64	55.71	382	382,364, 320,306,292,277,265, 201	6-methoxy-dihydronorchelerythrineN-oxide^a^or6-methoxy-dihydrorhoifoline B N-oxide^a^
65	56.32	322	322,307,292,279,264	Berberrubine^b^
66	59.18	336	336,321,305,293,278,264,250	8-O-demethyl-dihydrochelerythrine^a^
67	61.75	272	272,257,242,215,196,174,164,149,135	1,2-dihydroxy-3-methoxy-10-methyl-9-acridone
68	62.26	320	320,305,277,248	9,10-didemethylchelerythrine
69	63.48	334	334,318,304,290,276	Dihydrosanguinarine^b^
70	65.6	380	380,362,334,332,316,304,289,275	6-hydroxy-10-methylsanguinarine^b^

^a^firstly reported in the genus *zanthoxylum*

^b^identified by comparing with literatures

#### Benzophenanthridine type

Seven ZAs (peaks **31**, **48**, **51**, **54**, **60**, **62** and **68**) were identified and classified as benzophenanthridine type. With four benzene rings conjugated into the major skeleton, alkaloids of this type produced abundant fragments by neutral loss of substituents in the positive ESI-MS/MS [27].

Peaks **51**, **54**, **60** and **62** could be identified as 8-O-demethylchelerythrine, sanguinarine, nitidine and chelerythrine, respectively, by comparing their MS/MS spectra with the published literatures [11, 15, 26]. For peak **51**, the most intensive fragment at *m/z* 319 was observed due to the neutral loss of ∙CH_3_ from the molecular ion ([M + H]^+^ at *m/z* 334) at N moiety. The successive loss of CH_3_ (15 Da) and CO (28 Da) from the fragment ion at *m/z* 319 led to the fragments at *m/z* 304 and 291, respectively. Combined with the further loss of CO (28 Da) from fragment at *m/z* 304, peak **51** can be definitely identified as 8-O-demethylchelerythrine [27]. As to sanguinarine (peak **54**), its fragment ion at *m/z* 317 was produced by the loss of CH_3_ from the molecular ion ([M + H]^+^ at *m/z* 332). The most abundant fragment ion at *m/z* 274 was observed due to the neutral loss of CO from fragment ion at *m/z* 304. Therefore, peak **54** can be definitely identified as sanguinarine [11, 26, 27]. With same parent ion [M + H]^+^ at *m/z* 348, and the same fragment ions with relative intensity at *m/z* 332, 318, 304 and 290, peak **60** and **62** could be identified as nitidine or chelerythrine based on the investigation and comparison of their MS/MS spectra with those reported literatures [11, 15, 26, 27]. However, the distinguished retention time of peak **60** and **62**, which are 45.22 min and 48.20 min, can be used to differentiate them. Separated by Reversed-Phase Chromatography, the different retention time provided further evidence for identifying peaks **60** and **62** as nitidine and chelerythrine, respectively [15].

For peak **48**, its fragment ions at *m/z* 350, 320 and 292 were 2 Da more than fragment ions at *m/z* 348, 318 and 290 of nitidine. The different fragment ions at *m/z* 335 and 307 could be deduced by successive neutral loss of CH_3_ (15 Da) from N and CO (28 Da) from A ring of the fragment ion at *m/z* 350. Thus, peak **48** could be identified as fagaronine [25, 29]. With the same fragment ions as peak **48** at *m/z* 350, 335, 320, 307 and 292, the parent ion ([M + H]^+^) for peak **31** at *m/z* 512 indicated the presence of a glycoside. Thus, peak **31** was identified as fagaronine-3-glucoside [29]. In the MS/MS spectrum of peak **68**, fragment ions at *m/z* 305 and 277 were 30 Da less than those at m/z 335 and 307 of fagaronine. Thus, peak **68** could be proposed as 7,8-dimethylchelerythrine [27].

#### Dihydrobenzophenanthridine type

In this study, 12 peaks (peaks **28**, **30**, **33**, **43**, **56**, **58**, **61**, **63**, **64**, **66**, **69** and **70**) were identified and classified into dihydrobenzophenanthridine type. Compared with alkaloids of benzophenanthridine type, the N_7_ - C_8_ double bond of B ring was reduced to be a single bond in the skeleton of this type. Thus, the characteristic fragment ion was always observed to be [M + H-CH_4_]^+^ in the positive mode from the MS/MS spectra [27].

Compared the MS/MS data with those from authentic standards, peaks **43**, **61**, **63**, **69** and **70** were identified as 6,10-dihydroxyl-3- demethyldihydrobenzophenanthridinium, dihydrochelirubine, oxychelerythrine, dihydrosanguinarine and 6-hydroxy-10-methylsanguinarine [26], respectively. With [M + H]^+^ at *m/z* 384, peak **43** produced the most abundant fragment ion at *m/z* 352 by neutral loss CH_3_OH (32 Da) of from D ring, and the further successive loss of CH_3_ (15 Da) and OH (17 Da) led to fragment ions at *m/z* 337 and 320. Thus, peak **43** was identified as 6,10-dihydroxyl-3-demethyldihydrobenzophenanthridinium [26]. For peaks **61** and peak **63**, same molecular ion [M + H]^+^ at *m/z* 364 and fragment ions at *m/z* 348 and 334 were observed in the MS/MS spectra of these two peaks. However, in terms of the different fragments at *m/z* 332, 320, 304 and 290 for peak **61** and *m/z* 316, 306, 289 and 270 for peak **63**, peaks **61** and **63** were identified as dihydrochelirubine [19] and oxychelerythrine [26], respectively. For peak **69**, the most abundant fragment ion at *m/z* 318 was produced corresponding to the loss of CH_4_ from N moiety, due to the double bond on B ring reduced. With the same fragment ions at *m/z* 304 and 290 as peak **61**, the loss of CO (28 Da) from fragment ion at *m/z* 304 resulted in the different fragment at *m/z* 276. Therefore, peak **69** was assigned as dihydrosanguinarine [27].

As for peak **70**, a neutral loss of H_2_O from the molecular ion at *m/z* 380 [M + H]^+^ resulted in fragment ion at *m/z* 362. The cleavage of B ring led to the most abundant fragment at *m/z* 289 by loss of C_2_H_5_N (43 Da). Thus, peak **70** was definitely identified as 6-hydroxy-10-methylsanguinarine [26]. With the same molecular ion [M + H]^+^ at *m/z* 380 as peak **70**, fragment ions of peak **58** at *m/z* 349, 306 and 292 were observed. The neutral loss of substitute CO_2_ (44 Da) from fragment ion at m/z 364 yielded the most intensive fragment ion at *m/z* 320. Thus, peak **58** was deduced as 8-methoxy-dihydrochelerythrine or 8-methoxy-dihydronitidine [24]. In the positive mode, the alkaloid at 55.71 min (peak **64**) yielded fragment ions at *m/z* 364, 336, 306, 291 and 277, which were 2 Da more than fragment ions at *m/z* 362, 334, 304, 289 and 275 in peak **70**. Thus, this alkaloid (peak **64**) could be proposed as 6-methoxy-dihydronorchelerythrine N-oxide or 6-methoxy-dihydrorhoidoline B [26]. According to the MS/MS spectra of peak **33**, fragment ions at *m/z* 350 and 334 were produced by the loss of CH_4_ (14 Da) and CH_4_O (32 Da) from B ring, respectively. By B ring rearrangement and further neutral loss of C_2_H_4_O (44 Da), fragment ion at *m/z* 322 was produced. Thus, peak **33** was deduced as 6-methyl-5,6-dihydrofagaridine [30].

**Fig. 3:**
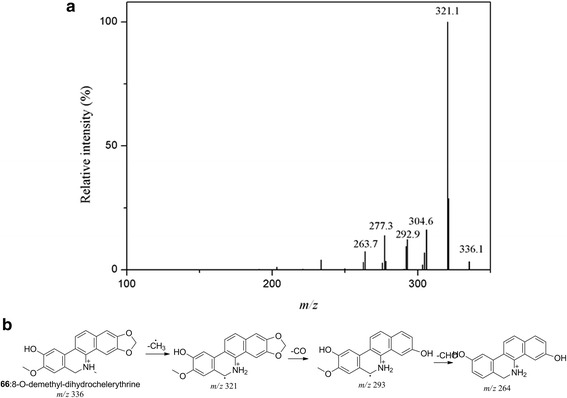
MS/MS spectra (**a**) and fragment pathways (**b**) of peak **66**

With molecular ion [M + H]^+^ at *m/z* 352 for peak **28** and 354 for peak **30**, same fragment ions at *m/z* 322, 308 and 280 were observed, while fragments at *m/z* 339 and 293 in the MS/MS spectra of peak **30** were 2 Da more than the fragment ions of peak **28** at *m/z* 337 and 291. The loss of H_2_O from fragment ion at *m/z* 337 resulted in fragment ion at *m/z* 319, indicating the presence of an OH on the parent molecular ion. In addition to the 2 Da discrepancy of the molecular and fragment ions, peak **28** and **30** were deduced as buegenine and 2-dimethoxy-3- hydroxybeugenine [31]. Peak **66** had [M + H]^+^ at *m/z* 336 and fragment ions at *m/z* 321, 293, 278 and 264 were 2 Da more than fragment ions at *m/z* 319, 291, 276 and 262 of 8-O-demethylchelerythrine (peak **51**) [27]. As the MS/MS spectra and proposed fragment pathways shown in Fig. 3, peak **66** was identified as 8-O-demethyl-dihydrochelerythrine [27]. As to peak **56**, the same fragment ions to peak **66** indicated the similarity of their structures. The loss of a glycosyl (162 Da) implied the compound to be an alkaloid glucoside. Hence, peak **56** had a molecular ion [M + H]^+^ at *m/z* 498 and was identified as 8-O-demethyl-dihydrochelerythrine-9-O-glucoside [27].

#### Berberine type

Compared with the benzophenanthridine type alkaloids, the structural skeleton of berberine type of alkaloids had double bond of C_5_ - C_6_ reduced. In the MS/MS spectra of berberine type of alkaloids, most fragment ions were successively generated by neutral loss of substituents [26]. Based on the reported literatures, five alkaloids (peaks **39**, **44**, **52**, **53** and **65**) were identified and classified into this group.

By comparing with the reported literatures, four alkaloids (peaks **44**, **52**, **53** and **65**) were identified as jatrorrhizine (peak **44**) [25], palmatine (peak **52**) [32, 33],berberine (peak **53**) [26, 27] and berberrubine (peak **65**) [26], respectively. In terms of peak **39**, fragment ions at *m/z* 368, 352, 338 and 310 were 30 Da more than fragment ions at *m/z* 338, 322, 308 and 280. The gap between the two peaks indicated the structural similarity and might be attributed to a CH_2_O (30 Da) lose. In addition to the investigation of substituents on this skeleton, such as 13-hydroxylberberruine and 13-methylberberine [26], C-13 was the most proposed position. Thus, peak **39** could be assigned as 10-hydroxy-2,3,9,12-tetramethoxy-jatrorrhizine [25].

#### Tetrahydroproberberine type

Alkaloids of tetrahydroberberine type produced their characteristic and most intensive fragment ions typically via Retro-Diels-Alder (RDA) cleavages [34]. Based on the detected MS/MS data, six alkaloids (peaks **23**, **26**, **27**, **40**, **45** and **47**) were identified and classified into this type.

Based on the reported MS/MS spectra, four alkaloids were identified as tetrahydropalmatine (peak **23**), N-methylcanadine or its isomers (peak **40** and **47**) and N-methyltetrahydropalmatine (peak **45**), respectively [25, 26]. In the MS/MS spectra of peak **23**, fragment ions at *m/z* 192 and 163 were observed due to the C ring cleavage. The further neutral loss of CH_2_ (14 Da) from fragment ion at *m/z* 163 led to the fragment ion at *m/z* 149. In addition, another abundant fragment ion at *m/z* 177 was produced by RDA cleavage of B ring. Thus, this alkaloid (peak **23**), with molecular ion [M + H]^+^ at m/z 356, was definitely identified as tetrahydropalmatine [25]. In terms of peaks **40** and **47**, the characteristic fragment ions at *m/z* 190 and 175 were 2 Da less than fragment ions at *m/z* 192 and 177 of peak **23**. With [M + H]^+^ at *m/z* 354, peaks **40** and **47** had same fragment ions and could be identified as N-methylcanadine or its isomers [26]. For peak **45**, the most abundant fragment ion at *m/z* 206 was 14 Da more than fragment ion at *m/z* 192 of peak **23**, which indicated the presence of a CH_2_ on A or B ring. Therefore, peak **45**, with parent ion [M + H]^+^ at *m/z* 370, was identified as N-methyltetrahydropalmatine [26].

**Fig. 4:**
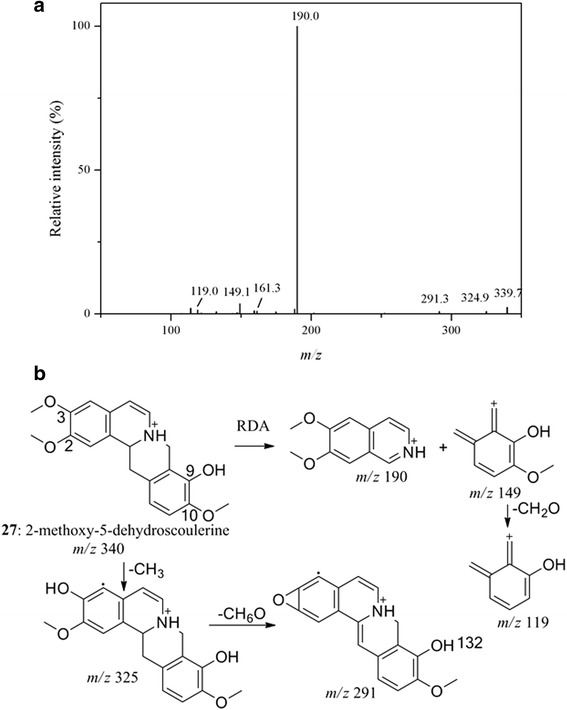
MS/MS spectra (**a**) and fragment pathway (**b**) of peak **27**

Peak **26** had the same fragment ions at *m/z* 192, 177 and 149 as peak **23**. The further cleavage of B ring from fragment ion at *m/z* 192 led to the distinguished fragment ion at *m/z* 151, which corresponded to a CH_3_ combined with N moiety. Thus, peak **26** with parent ion [M + H]^+^ at *m/z* 356 was identified as N-methyltetrahydrocolumbamine [35]. Peak **27** produced characteristic fragment ions at *m/z* 190 and 149 by RDA cleavage at C ring. As a result of further loss of CH_2_O (30 Da) from fragment ion at *m/z* 149, fragment ion at *m/z* 119 was observed. The successive neutral loss of CH_3_ and CH_6_O from the molecular ion [M + H]^+^at *m/z* 340 resulted in fragment ions at *m/z* 325 and 291. Therefore, peak **27** was deduced as 2-methoxy-5-dehydroscoulerine [36]. The MS/MS spectra and proposed fragment pathway of peak **27** was shown in Fig. 4.

#### Protopine type

Compared with tetrahydroproberberine type alkaloids, alkaloids of this type have carbonyl at C_14_, other than N_7_-C_14_. As for MS/MS spectra in ESI-MS/MS, major fragment ions were produced by the loss of H_2_O (18 Da) from parent ion [M + H]^+^and further RDA cleavage [27]. By comparing with reported literatures, peaks **9** and **42** were classified into protopine type and identified as hunnemannine [26] and α-allocryptopine [26, 37], respectively. Peaks **9** and **42** had the same fragment ions at *m/z* 206, 188 and 149, which were abundant and generated mainly by RDA cleavage. To distinguish the two peaks, fragment ion at *m/z* 338 of peak **9** yielded fragment ion at *m/z* 323 by neutral loss of CH_3_ (15 Da) from N. As to peak **42**, the loss of CH_4_O (32 Da) from fragment ion at *m/z* 352 led to fragment ion at *m/z* 320. Thus, peaks **9** and **42**, with molecular ion [M + H]^+^ at *m/z* 356 and 370, were identified as hunnemannine and α-allocryptopine, respectively.

#### Aporphine type

Due to the loss of substituents on A and D ring and N vicinal moieties through B ring cleavage, most abundant fragment ions of aporphine type were produced in the MS/MS spectra. By comparing with reported literatures, five peaks (peaks **11**, **20**, **29**, **32** and **34**) were classified into aporphine type and identified as magnoflorine [37], laurifoline [37], menisperine [37], 10-demethyl-magnoflorine [26] and Xanthoplanine [37], respectively.

With molecular ion [M + H]^+^ at *m/z* 342, fragment ions at *m/z* 282, 265, 237, 222 and 191 were observed both in the MS/MS spectra of peak **11** and **20**. Different fragment ion at *m/z* 233 was generated by the neutral loss of CH_4_O (32 Da) from fragment ion at *m/z* 265. Another fragment ion [M + H-C_2_H_7_N-2CH_4_O-CO]^+^ at *m/z* 205 was found to be 2 Da less than fragment ion at *m/z* 207 in the MS/MS spectra of peak **11**. Therefore, peaks **11** and **20** were identified as magnoflorine and laurifoline by comparing the fragments relative intensity and differences, respectively [37]. As to peaks **29** and **34**, same fragment ions at *m/z* 311, 296, 219, 192 and 177 were observed. With intensive and same fragment ions at *m/z* 279, 264, 248 and 236, peak **29** had retention time at 21.71 min and was identified as menisperine [37]. For peak **34**, the loss of N vicinal moieties and substituents from A and D ring led to fragment ions at 280, 265, 253 and 237. Thus, peak **34** had retention time at 24.70 min and was identified as Xanthoplanine [37]. In reference to peak **32,** fragment ions at *m/z* 251, 208 and 191 were 14 Da less than fragment ions at *m/z* 265, 222 and 205 in the MS/MS spectra of peak **20**. The most intensive fragment ion at *m/z* 121 indicated the presence of 2OH on D ring. Thus, peak **32** was proposed as 10-demethyl-magnoflorineby comparing with the reported data [26].

#### Benzyltetrahydroisoquinoline type

Biosynthesis analysis of ZAs provided evidence of benzyltetrahydroisoquinoline type alkaloids as precursor of alkaloids, such as alkaloids of benzophenanthridine type, berberine type and aporphine type [38]. As to the MS/MS spectra of benzyltetrahydroisoquinoline type of alkaloids, fragment ions with most abundant intensity were produced by the neutral loss of N vicinal moieties and substituents on iso-quinoline ring [27]. Among the detected alkaloids, ten peaks (**4**, **6**, **7**, **8**, **12**, **15**, **18**, **21**, **22** and **36**) were identified and classified into benzyltetrahydroisoquinoline type.

By comparing with published MS/MS data, peaks **7**, **8**, **18**, **21** and **36** were identified. Same fragment ions and relative intensity were observed in the MS/MS spectra of peaks **7**, **21** and **36**. The loss of benzyl from B ring led to fragment ions at *m/z* 209 and 107. Due to the cleavage of B ring, fragment ions at *m/z* 166 and 137 were also observed. The successive loss of C_2_H_7_N (45 Da) and CH_4_O (32 Da) from the parent ion [M + H]^+^ at *m/z* 314 generated fragment ions at *m/z* 269 and 237. Thus, the three compounds (peaks **7**, **21** and **36**) were identified as magnocurarine or its isomers [37]. In reference to peak **6**, the alkaloids had molecular ion [M + H]^+^ at *m/z* 476 and same fragment ions with peak **7**. The mass gap between parent ion and fragment ion at *m/z* 314 indicated the possible presence of a glycosyl. Therefore, with retention time at 11.67 min, peak **6** was deduced as magnocurarine-6-glucoside [37]. Due to the loss of benzyl in the MS/MS of peak **8**, the characteristic fragment ion at *m/z* 107 was observed. Moreover, the same fragment ions to peak **7** at *m/z* 269 and 237 showed the structural discrepancy at N vicinal moieties. Thus, peak **8**, with the molecular ion at *m/z* 286, was identified as 7-methoxyhigenamine based on its reported mass data [37]. As to peak **18**, the loss of benzyl and its substituents yielded fragment ions at *m/z* 192 and 137, and the further loss of CH_3_ (15 Da) from isoquinoline ring produced the fragment ion at *m/z* 177. Due to the loss of substituents and N vicinal moieties, fragment at *m/z* 207 [M + H-C_7_H_7_O_2_]^+^ generated fragment ions at *m/z* 175, 143 and 115 by similar fragment pathway with peak **8**. Thus, peak **18**, with molecular ion [M + H]^+^ at *m/z* 330, was identified as reticuline [37].

**Fig. 5:**
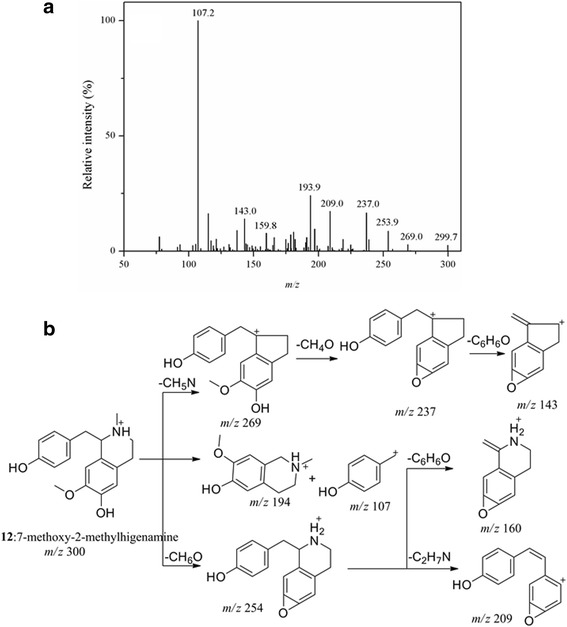
MS/MS spectra (**a**) and fragment pathway (**b**) of peak **12**

With retention time at 12.28 min, peak **12** had fragment ions at *m/z* 269, 254, 237, 160, 143 and 107, which was almost the same to peak **8**. In contrary to the loss of NH_3_ (17 Da), fragment ion at *m/z* 269 was produced by the loss of CH_3_-NH_2_ (31 Da). To display the fragment pathway of this type of alkaloids in detail, the MS/MS spectra and fragment pathway of peak **12** was shown in Fig. 5. Thus, peak **12**, with the molecular ion [M + H]^+^ at *m/z* 300, was deduced as 7-methoxy-2-methylhigenamine [37]. The same fragment ions of MS/MS spectra from peak **4** and peak **12** indicated the structure similarity between the two alkaloids. In addition to the molecular ion [M + H]^+^ at *m/z* 462, fragment ion at *m/z* 300 was produced by the loss of a glycosyl. Thus, peak **4**, with retention time at 8.83 min, was deduced as 7-methoxy-2- methylhigenamine-4′-glucoside or 7-methoxy-2-methylhigenamine-6-glucoside by comparing with the mass data of 7-methoxy-2-methylhigenamine [37]. For the fragment ions of peak **15**, the characteristic loss of benzyl and its substituents led to fragment ions at *m/z* 207 and 137. Moreover, the N vicinal moiety loss generated fragment ion at *m/z* 301, of which the further substituents loss yielded fragment ions at *m/z* 269 [M + H-C_2_H_5_N-CH_4_O]^+^ and 239 [M + H-C_2_H_5_N-CH_4_O-CH_2_O]^+^. Therefore, peak **15** was speculated as isotembetarine or xylopinidine [25, 35]. In reference to peak **22**, the characteristic loss of benzyl generated the fragment ions at *m/z* 221 and 137. The molecular ion [M + H]^+^ at *m/z* 358 and fragment ion at *m/z* 221 in the MS/MS spectra were 14 Da more than [M + H]^+^ at *m/z* 344 and fragment ion at *m/z* 207 of peak **15**, respectively. The mass gap between the two compounds indicated a methyl substituent discrepancy on the isoquinoline ring. Thus, with retention time at 19.92 min, peak **22** could be deduced as 8-methoxy-isotembetatrine [25].

#### Quinoline or Isoquinoline type

Ten peaks (**1**, **2**, **3**, **5**, **13**, **19**, **24**, **35**, **37** and **67**) were identified and classified into quinoline or isoquinoline type. In addition to the relative fragment ions intensity of peak **1**, fragment ions at *m/z* 177 and molecular ion [M + H]^+^ at *m/z* 192 were also observed in the MS/MS spectra of tetrahydropalmatine. The successive neutral loss of substituents led to fragment ions at *m/z* 162 [M + H-CH_2_O]^+^ and 148 [M + H-CH_2_O-CH_2_]^+^. Therefore, peak **1** was deduced as 6,7-dimethoxy- 3,4-dihydroisoquinoline [25]. With retention time at 6.11 min, fragment ions of peak **2** at *m/z* 190, 162 and 132 were observed due to the successive loss of CH_4_ (16 Da),CO (28 Da) and CH_3_OH (31 Da) from parent ion. The further loss of N moiety and substituent from fragment ion at *m/z* 190 led to fragment ions at *m/z* 173 and 145. Therefore, peak **2**, with molecular ion at *m/z* 206, was tentatively identified as edulitine [39]. For peak **3**, the cleavage of isoquinoline ring resulted in fragment ions at *m/z* 169 and 145 by the loss of N vicinal moiety from fragment ion at *m/z* 167. Intensive fragment at *m/z* 117 was generated by further loss of CO (28 Da) from fragment ion at *m/z* 145. As a result, peak **3**, with retention time at 7.39 min, was tentatively assigned as thalifoline [40]. Compared with peak **3**, the discrepancy fragment ion at *m/z* 149 obtained from peak **5** explored the differences of the two alkaloids on the isoquinoline ring. Taking the neutral loss of NH_3_ (17 Da) from fragment ion at *m/z* 162 into consideration, the lack of substituent on N was proved. Thus, peak **5**, with molecular ion [M + H]^+^ at *m/z* 194, was deduced as Des-N-methyl-thalifoline [40].

As to peak **67**, the successive loss of CH_3_ (15 Da),CH_2_O (30 Da) and C_6_H_4_ (76 Da) from parent ion led to fragment ions at *m/z* 257, 242 and 196, and fragment ions at *m/z* 164 and 149 were produced by successive loss of CH_3_OH (32 Da) and CH_3_ (15 Da) from fragment ion at *m/z* 196. Therefore, peak **67**, with molecular ion [M + H]^+^ at *m/z* 272, was deduced as 1,2-dihydroxy-3- methoxy-10-methyl-9-acridone [41]. With same fragment ions as peak **67,** peak **13** had molecular ion [M + H]^+^ at *m/z* 434, which revealed the presence of a glycosyl in this alkaloids. Thus, with retention time at 15.85 min, peak **13** was deduced as 2-dihydroxy-3-methoxy-10-methyl-9-acridone-1-glucoside or 1-dihydroxy-3-methoxy-10-methyl-9-acridone-2-glucoside [41]. For peak **24**, RDA cleavage of C ring produced the most intensive fragment ion at *m/z* 188, and the further loss of CO (28 Da) yielded fragment at *m/z* 160. In addition, fragment ions at *m/z* 134 and 106 were observed due to the cleavage of B ring and substituent loss. Thus, with molecular ion [M + H]^+^ at *m/z* 260, peak **24** could be tentatively identified as ribalinine [42]. Another alkaloid (peak **35**) shared same fragment ions to peak **24**. Fragment ion at *m/z* 260 indicated a loss of a glycosyl from parent ion [M + H]^+^ at *m/z* 422. Thus, with retention time at 25.33 min, peak **35** could be tentatively deduced as ribalinine 3′-glucoside [42]. Peak **19** had the same fragment ions at *m/z* 242, 188 and 160 as peak **24**, indicating the structure similarity of the two compounds. The cleavage of C ring led to fragment ions at *m/z* 218 [M + H-C_4_H_8_O]^+^ and 160 [M + H-C_6_H_10_O_3_]^+^, which confirmed the difference on C-4′. Therefore, with molecular ion [M + H]^+^ at *m/z* 290, peak **19** could be deduced as 4′-methoxy-ribalinine [42]. Peak **37** was detected at 25.92 min, and displayed same fragment ions with peak **19**. As a result of the loss of glycosyl, fragment ion at *m/z* 290 was observed. Thus, peak **37**, with molecular ion [M + H]^+^ at *m/z* 452, was deduced as 4′-methoxy-ribalinine-3′-glucoside [42].

### Comparison of ZAs detected among the three species

**Fig. 6:**
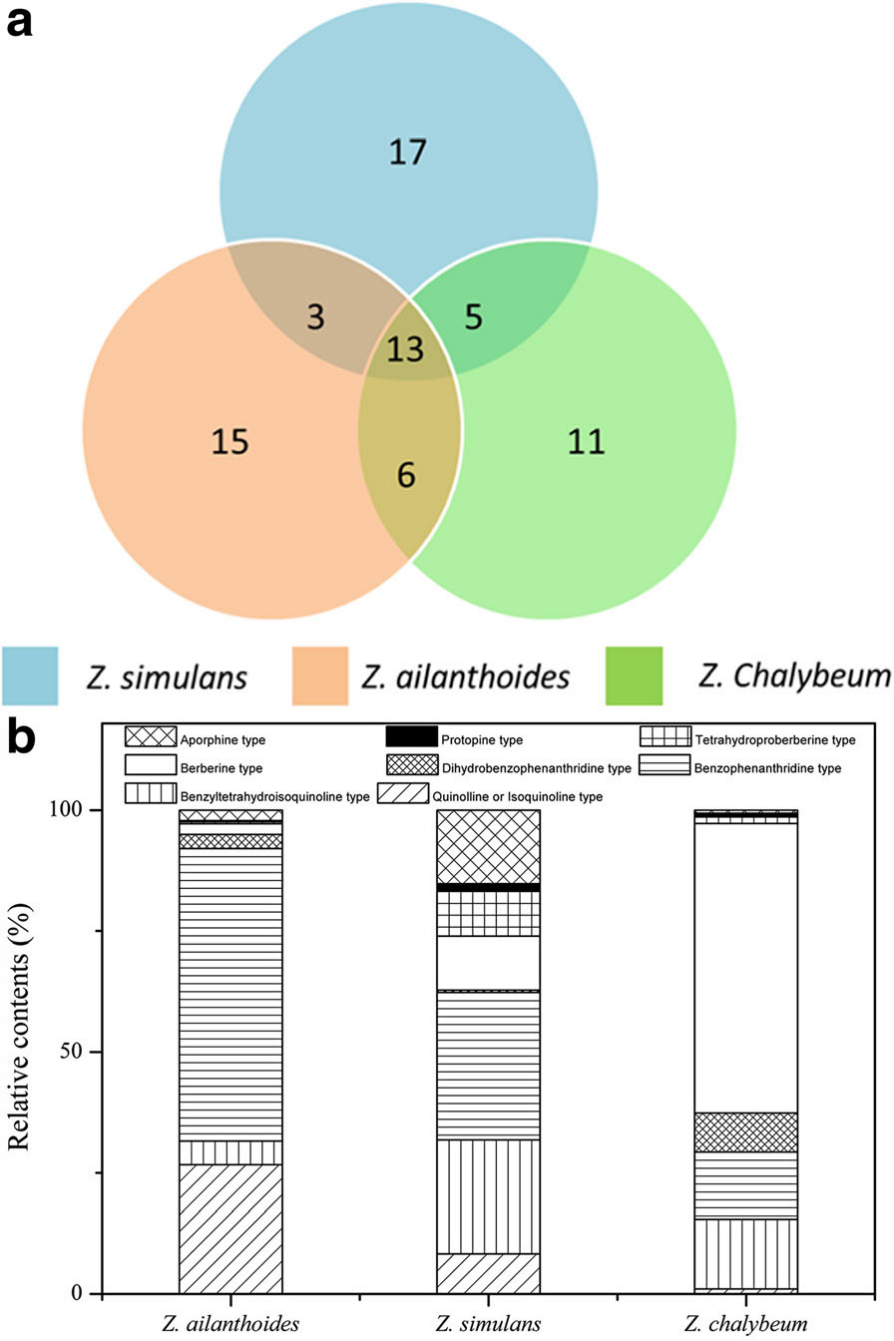
Qualitative (**a**) and quantitative (**b**) differences of ZAs from *Z. ailanthoides*, *Z. simulans* and *Z. chalybeum*

The fingerprinting profiles of ZAs in Fig. 1 showed a preliminary difference in the alkaloids among the three species (*Z. ailanthoides*, *Z. simulans* and *Z. chalybeum*). Since both qualitative and quantitative information may help to understand the most bio-active and potential alkaloids, the common and unique peaks for the three species were summarized in Fig. 6a. As a result, 13 common peaks **(**i.e., **3**, **5**, **11**, **17**, **18**, **34**, **39**, **42**, **46**, **51**, **62**, **68** and **70**) were found in the extracts of all the three *Zanthoxylum* species, while some unique peaks were also observed in specific species, which are 17 peaks in *Z. simulans*, 15 peaks in *Z. ailanthoides* and 11 peaks in *Z. chalybeum*, respectively. Moreover, three alkaloids (peaks **7**, **14** and **22**) were only found in *Z. ailanthoides* and *Z. simulans*, six alkaloids (peaks **2**, **21**, **28**, **30**, **60** and **64**) only in *Z. ailanthoides* and *Z. chalybeum*, and five alkaloids (peaks **6**, **15**, **16**, **47** and **53**) only in *Z. simulans* and *Z. chalybeum*. To some extent, these unique peaks may be used as chemical markers to differentiate these three species for quality control purposes.

Based on the alkaloids identification, the peak areas of each type of alkaloids from the three species (*Z. ailanthoides*, *Z. simulans* and *Z. chalybeum*) were summarized to explore quantitative differences of the ZAs, as shown in Fig. 6b. For alkaloids from *Z. ailanthoides*, benzophenanthridine type (60.52%) and quinoline or isoquinoline type (20.66%) were the most abundant alkaloids, followed by benzyltetrahydroisoquinoline type (4.88%), dihydrobenzophenanthridine type (2.94%), berberine type (2.17%), aporphine type (2.15%), tetrahydroproberberine type (0.42%) and protopine type (0.25%). For *Z. simulans*, benzophenanthridine type and benzyltetrahydroisoquinoline type contributed to 30.52% and 23.49%, followed by aporphine type (15.22%), berberine type (11.10%), tetrahydroproberberine type (9.28%), quinoline or isoquinoline type (8.30%), protopine type (1.59%) and dihydrobenzophenanthridine type (0.49%), respectively. For the alkaloids in *Z. Chalybeum*, the major alkaloids were in protopine type at relative content of 59.88%, together with benzyltetrahydroisoquinoline type at 14.42%, benzophenanthridine type at 13.84%, dihydrobenzophenanthridine type at 8.09%, tetrahydroproberberine type at 1.35%, quinoline or isoquinoline type at 1.02%, protopine type at 0.76% and aporphine type at 0.64%, respectively. Notably, based on the analysis of composition and contents of ZAs in the three species, alkaloids of benzophenanthridine type were found to be the most common ZAs, which accounts for over 14% in all the three species.

### Anti-proliferative activities of ZAs against carcinoma cells

**Fig. 7:**
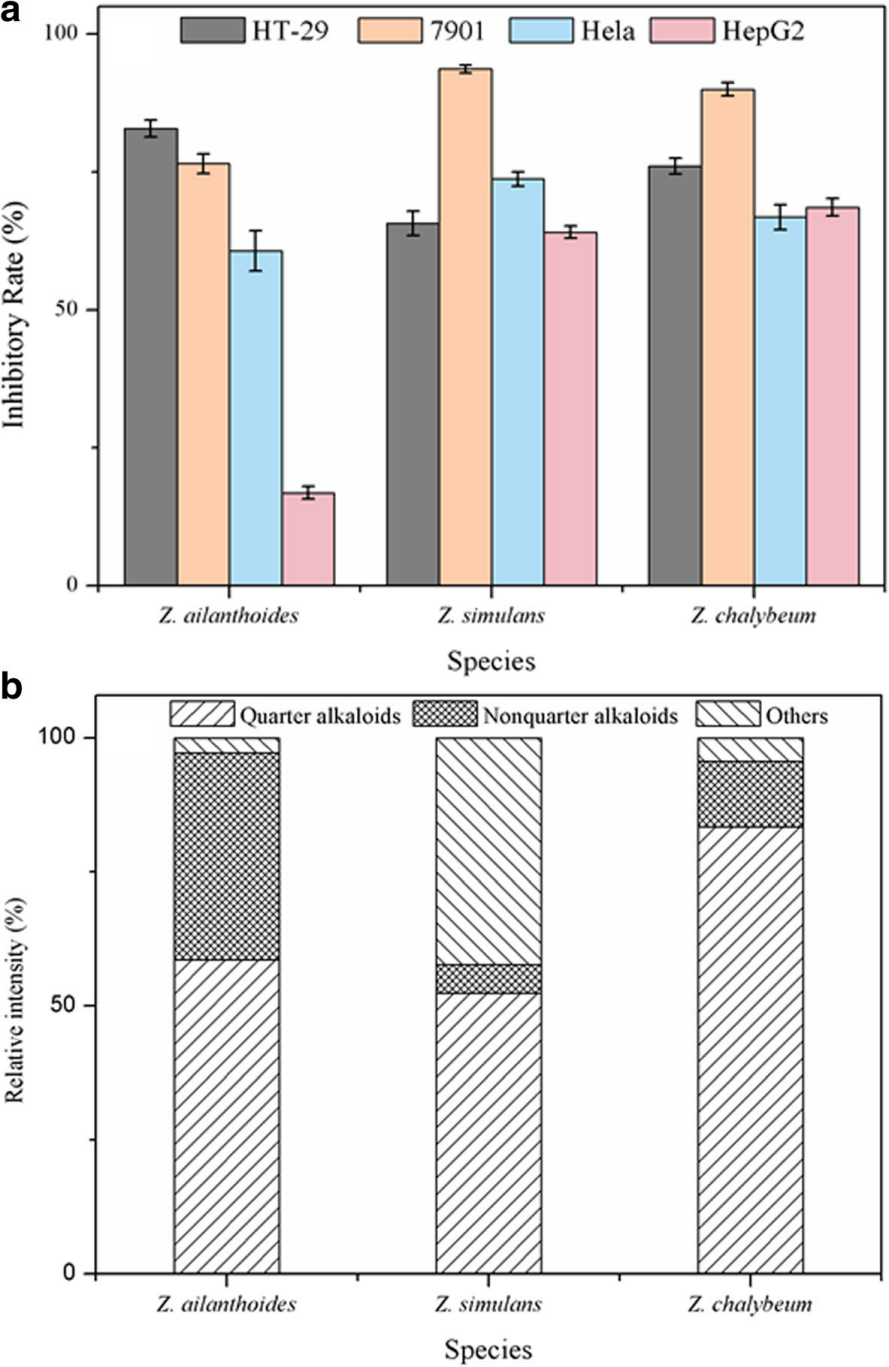
Inhibitory rates against four different cancer cell lines (**a**) and relative contents of quaternary alkaloids (**b**) of *Z. ailanthoides*, *Z. simulans* and *Z. chalybeum*

To explore the correlation between ZAs and their potential bioactivities, in vitro inhibitory activity tests against cancer cells have been performed on the crude extracts of alkaloids from the three *Zanthoxylum* species, using Sulforhodamine B (SRB) method to test four cancer cells, i.e., human gastric cancer cells (SGC-7901), cervical cancer cells (Hela), human colorectal adenocarcinoma cells (HT-29) and human hepatocyte carcinoma cells (Hep G2). As shown in Fig. 7, ZAs showed remarkably proliferative inhibition to all the four cancer cell lines, which ranged from 60.71 to 93.63% at a concentration of 200 μg/mL. An exception was found on the crude alkaloids of *Z. ailanthoides* with Hep G2 inhibitory rate at 16.83%. To some extent, SGC-7901 seemed to be more sensitive to the ZAs than the other three cancer cells.

It was well documented that the cancer cell inhibitory rate was not only relevant to the composition and content of the testing chemicals, but also to the water solubility of the chemicals [43], which inspired us to focus on the water-soluble ZAs. Quaternary alkaloids in *Zanthoxylum*genus have been recognized as promising anti-cancer candidates by penetrating through the carcinoma cell membrane [44] and attracting the negative electron on DNA [45]. Therefore, in this study, the percentage of quaternary alkaloids from the three *Zanthoxylum* species has been further evaluated for their contribution to the cancer cell inhibitory rate. As a result, with positive charge on the molecule, 27 ZAs have been classified into quaternary alkaloids from the three *Zanthoxylum* species, i.e., peaks **6**, **7**, **11**, **15**, **20**, **21**, **22**, **26**, **29**, **31**, **32**, **34**, **36**, **39**, **40**, **44**, **45**, **47**, **48**, **51**, **52**, **53**, **54**, **60**, **62**, **65** and **68**, which belongs to benzophenanthridine type, aporphine type, berberine type, benzyltetrahydroisoquinoline type and tetrahydroproberberine type according to their structural skeleton. As shown in Fig. 7b, other than non-quaternary and unidentified alkaloids, the percentages of quaternary alkaloids were 83.4%, 58.6%, 52.4% for *Z. Chalybeum*, *Z. ailanthoides*, *Z. simulans*, respectively. Since high content of quaternary alkaloids and high cancer cell inhibitory rates were simultaneously found on bioactivity tests of ZAs, the quaternary alkaloids could be proposed as the most potential bioactive compounds in the genus *Zanthoxylum*, which will provide great guidance to the further exploitation of this Chinese herb medicine as new drugs or healthcare products.

## Conclusions

Based on the HPLC-UV/ESI-MS/MS method developed in this work, a fingerprinting comparison was conducted on ZAs from *Z. ailanthoides*, *Z. simulans* and *Z. chalybeum*. As a result, remarkable differences were shown on the fingerprints in both the compositions and contents of alkaloids among the three *Zanthoxylum* species. In more details, a total of 70 peaks corresponding to alkaloids were detected, of which 58 peaks were identified by comparing their MS/MS spectra with the reported literatures. More interestingly, 27 quaternary alkaloids and 6 alkaloid-glycosides were reported for the first time. Moreover, carcinoma cell inhibitory rates to four cancer cell lines were investigated to evaluate the contribution of some distinct compositions. Consequently, quaternary alkaloids of ZAs were proposed as the most potent proliferative inhibitors against cancer cells and crude ZAs showed more sensitive to SGC-7901 cells than the other cancer cells. To the best of our knowledge, this work provides the most details on ZAs and their potential activities, and offered valuable information to further explore *Zanthoxylum* alkaloids in this genus as new healthcare products in the near future.
